# Replicating replicability modeling of psychology papers

**DOI:** 10.1073/pnas.2309496120

**Published:** 2023-08-07

**Authors:** Aske Mottelson, Dimosthenis Kontogiorgos

**Affiliations:** ^a^Department of Digital Design, IT University of Copenhagen, 2300 Copenhagen, Denmark; ^b^Department of Computer Science, Humboldt University of Berlin, 10099 Berlin, Germany; ^c^Science of Intelligence, Research Cluster of Excellence, 0587 Berlin, Germany

Youyou et al. (1) estimated the replicability of more than 14,000 psychology papers using a machine learning model, trained on main texts of 388 replicated studies. The authors identified mean replicability scores of psychological subfields. They also verified the causality of the model predictions; correlations between model predictions and study details the model was not trained on (i.e., P value and sample size) were reported.

In attempting replication, we identified important shortcomings of the approach and findings. First, the training data contain duplicated paper entries. Second, our analysis shows that the model predictions also correlate with variables that are not causal to replicability (e.g., language style). These issues impede the validity of the model output and thereby paint an erroneous picture of replication rates of the psychological science. In this letter, we attempt to mitigate these issues and nuance the findings of the original paper.

## 1. Replication

### A. Direct Replication.

The authors agreed to share data and parts of their Java modeling code, and we attempted to replicate their findings using Python. The replication was partially successful; implementing the described model and evaluations yielded a small performance decrease (see variations of results in Table 1).

**Table 1.** Comparing evaluations of the original study, a direct replication, and an improved replication

**Model** Tokenizer Model	Youyou et al. TF-IDF, word2vec Random forest, Logistic regression	Direct replication TF-IDF, word2vec Random forest, Logistic regression	Improved replication TF-IDF Random forest
**Data**			
Duplicate DOIs	37	37	0
Ambiguous labels	3	3	0
Sample size	388	388	348
**Performance**			
AUC	0.74	0.68	0.76
Binary accuracy	68%	63%	70%
**Correlations**			
P value	−0.42^*^	−0.23^*^	−0.23^*^
Sample size	0.31^*^	0.46^*^	0.44^*^
Number of words		0.24^*^	0.27^*^
Number of nouns		0.29^*^	0.32^*^
Linguistic features^A^		34%	40%

^*^denotes a P value below 0.05.

^A^Percentage of P values below 0.05 for correlations of 220 lftk features with model predictions.

### B. Dataset Issue.

We identified 37 duplicate DOIs (out of 388) and three papers with conflicting replication labels (e.g., both “yes” and “no” labels from multiple replications). These issues render the reported metrics of the original evaluation problematic.

### C. Improved Replication.

**Fig. 1.**
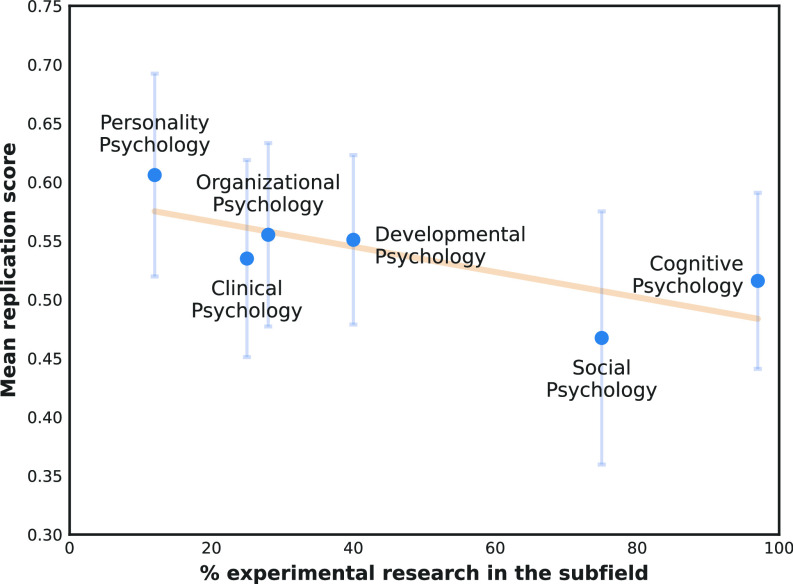
Percentage of experimental research in each psychology subfield and the subfield’s mean replication score. Error bars show SDs.

We attempted to mitigate the identified data issue. Our improved replication was trained using a similar approach as the one originally proposed, but duplicates and entries with ambiguous labels were removed. We furthermore optimized model parameters using grid search, omitted the use of stop words, and employed TF-IDFs. The accuracy of the model is slightly higher than the original (Table 1). Fig. 1 shows the recomputed estimated replication rates of psychological subfields, with a notable increase in the mean replication rate for Developmental Psychology.

## 2. Modeling Replicability

We have corrected the data issues in the original modeling work and nuanced replication estimations. Nevertheless, there are other issues which condition that results should be interpreted with caution. As psychological subfields are foremost determined by journal, it implies that publication cultures (e.g., prestige, language style, and textual restrictions) of individual journals will influence the replicability estimates of subfields when training on paper manuscripts.

### Causal Factors.

Youyou et al. (1) reported that model outcomes correlated with P values and sample sizes, underlining the causality of model outputs to replicability, as such content was removed from the training data. Nonetheless, we also identify correlations to factors unrelated to replicability, such as number of nouns, r(98)=0.29,P=0.003. Of 220 commonly used linguistic features (2), 75 significantly correlated to replication estimations. When correcting for multiple comparisons, five features of the domains surface, syntax, and discourse significantly correlated to model estimations, such as the number of named entities, r(98)=−0.45,P<0.0001. Such correlations suggest that the model learns from language style, which may vary systematically between subdisciplines of psychology.

## Data Availability

Code and noncopyrighted data are available without reservation at https://osf.io/rj2tk/ (3).
